# The Impact of Early Life Bilateral Salpingo‐Oophorectomy on the Nucleus Basalis of Meynert

**DOI:** 10.1002/alz70856_106937

**Published:** 2026-01-10

**Authors:** Zaki Ul Haq, Noelia Calvo, Laura Gravelsins, Alana Brown, Sophia Zhao, Nicole J Gervais, Annie Duchesne, Shreeyaa Ramana, Natasha Rajah, Cheryl Grady, Gillian Einstein

**Affiliations:** ^1^ University of Toronto, Toronto, ON, Canada; ^2^ Centre for Addiction and Mental Health, Campbell Family Mental Health Research Institute, Toronto, ON, Canada; ^3^ Rotman Research Institute, Baycrest Academy for Research and Education, Toronto, ON, Canada; ^4^ University of Groningen, Groningen, Groningen, Netherlands; ^5^ University of Northern British Columbia, Prince George, BC, Canada; ^6^ Université du Québec à Trois‐Rivières, Trois‐Rivières (Quebec), QC, Canada; ^7^ Toronto Metropolitan University, Toronto, QC, Canada; ^8^ Department of Psychiatry, McGill University, Montréal, QC, Canada; ^9^ Baycrest Academy of Research and Education, Toronto, ON, Canada; ^10^ Women's College Research Institute, Toronto, ON, Canada; ^11^ Rotman Research Institute, Toronto, ON, Canada

## Abstract

**Background:**

Alzheimer's disease (AD) disproportionately affects women. Women with early‐life bilateral salpingo‐oophorectomy (BSO) have a yet higher risk of late‐life AD. The cholinergic pathway in the basal forebrain, comprising cholinergic nuclei 1, 2, and 3 (Ch123) and the nucleus basalis of Meynert (NBM, i.e., Ch4), are sensitive to estradiol and its atrophy is a marker for AD risk. We wondered whether BSO‐mediated estradiol depletion would affect its structure thereby helping to elucidate these women's trajectory toward late‐life AD risk.

**Methods:**

Participants included women with early BSO taking estradiol therapy (BSO_ET_; *n* = 24, *M±SD* age at scan = 45.1±5.2), women with early BSO without ET (BSO; *n* = 27, *M±SD* age = 47.3±6.5) and age‐matched controls (AMC; *n* = 22, *M±SD* age = 44.0±5.5) from Toronto and Montreal, Canada. T1‐weighted MRI scans (Siemens Prisma/Philips 3T) were preprocessed using the Computational Anatomy toolbox (CAT12). Cholinergic volumes (Ch123, NbM) were identified using cytoarchitectonic probabilistic maps from the JuBrain Anatomy toolbox. Group comparisons were assessed using bivariate analysis (ANOVA, or *X* ^2^). Partial least squares correlation (PLSC) was then used to examine associations only in the women with BSO between basal forebrain volumes and BSO‐related variables (e.g., time since BSO, estradiol therapy) and hormone levels (estradiol, progesterone). Statistical strength of the model was assessed using resampling methods (permutation test and bootstrap).

**Results:**

Groups did not differ significantly in age or education. Women with BSO_ET_ had higher estradiol levels at scan (M±SD = 38.5±36.5) than AMC (36.2±28.1) and BSO (17.6±8.1). They also had higher progesterone levels at scan (BSO_ET_: M±SD = 8.0±17.0) than both groups (AMC: 3.3±5.8; BSO: 1.9±3.8). Bivariate analysis did not find significant differences in Cholinergic volumes among the three groups. However, exploratory PLSC of BSO groups identified one latent variable explaining 95.2% of the covariance (*p* = 0.006) which linked older age at scan and more time since BSO (irrespective of estradiol therapy) to smaller NbM volumes bilaterally.

**Conclusion:**

Preliminary findings suggest that aging and early‐BSO may influence basal forebrain structure. Future work should disentangle age/time‐since‐BSO from estradiol loss. It might also investigate further memory circuit disruption by examining surrounding cholinergic white matter pathways further identifying early AD biomarkers.

